# Dental research in Spain. A bibliometric analysis on subjects, authors and institutions (1993-2012)

**DOI:** 10.4317/medoral.20756

**Published:** 2016-01-31

**Authors:** Felipe Bueno-Aguilera, Evaristo Jiménez-Contreras, Cristina Lucena-Martín, Rosa Pulgar-Encinas

**Affiliations:** 1 Department of Stomatology, Faculty of Dentistry, Campus de Cartuja, Ed. Máximo, Granada University, 18071 Granada, Spain; 2 EC3 Research Group ,Department of Information Science, Faculty of Communication, Campus Cartuja, Ed. Máximo, Granada University, 18071 Granada, Spain

## Abstract

**Background:**

Bibliometrics is defined as the use of statistical methods in the analysis of a body of literature to reveal the historical development of subject fields and patterns of authorship, publication, and use. Our objective was to characterize Spanish scientific output in Dentistry through the analysis of Web of Science database in a 20-year period. By means of a bibliometric study documents were statistically analyzed using indicators that showed quantitative and qualitative aspects of the production. Specifically, time course of the scientific production within the time span was analysed, as were the journals where the article was published and the categories of Journal Citation Reports (JCR) in which they belong, thematic areas, authorship, and finally authors and institutions with the highest production in Spain.

**Material and Methods:**

By means of the design of a specific search strategy previously described in the scientific literature, we recovered all citable documents about Dentistry signed by Spanish researchers and included in the WoS database between 1993 and 2012.

**Results:**

A total of 3006 documents fulfilled the search criteria, of which 2449 (81.5%) were published in journals within the category Dentistry Oral Surgery and Medicine and 557 (18.5%) within other categories of the JCR. During the four quinquenniums studied, the production increased quantitatively (8.6-fold) and qualitatively. Finally, the universities of Granada and Complutense of Madrid were the institutions with the highest production and most prolific authors.

**Conclusions:**

The Spanish dental production sharply increased in the last two decades, reaching quantitative and qualitative levels similar to those of the other medical specialties in the country.

**Key words:**Dental research, dentistry, publications, Journal impact factor, bibliometrics, biomedical research, Spanish dental production.

## Introduction

Scientometrics measures the advance of science, enabling us to characterize the output of authors, institutions, and countries, as well as a defined specialty or thematic area. Such studies provide useful and objective information for planning research and develop ment programs, and for optimizing human resources and materials to fit with the real needs of the scientific community.

In this sense, there is extensive literature concerning how world scientific output has steadily increased during recent decades (1-3). This has also occurred in Spain for both the total production (3,4) and biomedical output (5). Recent studies have shown how this trend also holds for Spanish dental research, showing what this research quantitatively represents in a global context (6,7). However, to date no thorough analysis has been undertaken for the scientific dental research in Spain, whether in relation to the size, time course, thematic areas, the most productive authors and institutions, or how these factors interact.

Consequently, our aim is to analyse the scientific dental research output in Spain for the period 1993-2012. In this first study, we characterize the production, analysing how it has progressed quantitatively and qualitatively over the timespan studied. We consider the journals where this research has been published and the respective categories of Journal Citation Reports (JCR), the thematic areas (specialties) and finally, the most productive authors and institutions in the country.

## Material and Methods

The study was approved by the ethical commission of Granada University. The database selected to analyse Spanish dental-research output was the Thomson Reuters Web of Science (WoS). The research spanned more than 20 years, with references published in any language. For analysis, only citable documents were taken into account (articles, reviews, letters, and notes) and, for the time course, we considered four periods: 1993-97, 1998-2002, 2003-07 and 2008-12. The searches were made between May and June of 2013. The documents were retrieved in two stages: first, all documents published in the journals included in Dentistry, Oral Surgery and Medicine (DOSM) category of the Journal Citation Reports (JCR) database for the four 5-year periods studied were directly downloaded. Secondly, to retrieve dental documents published in any other journals included in any other categories of the JCR database, hereafter called non-DOSM, we used the methodology previously described by Pulgar *et al*. (7). Specifically, we defined “dental literature” as any scientific document with content related to dental subjects (e.g. “lip” could be a dental subject but also dermatological) and produced by a dental institution, identified by its institutional address. Therefore, a thematically mixed search strategy, as well as institutional one, was designed. The keywords for the thematic search and institutional search were the same as used by Pulgar *et al*. (7). Afterwards, bibliographic software was used and, in non-DOSM databases, duplicated records were eliminated.

From the database generated, we extracted the documents related to Spain by means of a search in the fields “addresses” and “reprint address” with the term “Spain”. This ensured that the documents were signed by at least one Spanish institution or the Spanish division of an international one.

Once the database was filtered, we identified the JCR categories to which each document belonged. To assign a document to a specific category, we used a multi-count approach so that each one was assigned to all categories where the journal was included. Therefore the sum of documents belonging to each category was greater than total. We distinguished between the journals belonging to DOSM and the rest, and the documents appearing in both, were counted only in the first one.

In the production thematic analysis, we ascribed each article to one or several specialties of Dentistry. To do so, we followed the criterion described in Pulgar *et al*. (7), allocating papers to subject areas based on dental specialties recognized by the American Dental Association (ADA). These are: Endodontics, Orthodontics and Dentofacial Orthopedics, Oral and Maxillofacial Pathology, Pediatric Dentistry, Periodontics, Prosthodontics, Dental Public Health, Oral and Maxillofacial Radiology, and Oral and Maxillofacial Surgery. General Dentistry was also included and represents dental fields not included in the above-mentioned specialties, i.e. those basically related to Operative Dentistry. Also, we included Dental Materials and Implants because they are interest areas with a well-defined body of evidence based on scientific and clinical knowledge. For the documents of the databases (both DOSM as well as non-DOSM) to be associated with the dental specialties considered, we created an allocation strategy of keywords based on the previously selected keywords (229 descriptors) in the MESH (7). For an article covering various themes, we used several of the truncated terms associated with a number of specialties to label it, thus offering us information on how these different disciplines are related within Dentistry.

The authorship analysis was conducted in two stages. First, establishing the average signers per article over the period; and second, identifying the most productive authors. For thorough results, we needed to normalize the names of the potential producers. This process required a manual search for each one.

To identify the most productive institutions in dental research in Spain, in this case, we filtered the institutional affiliation field one by one to eliminate spelling mistakes and normalize the different names given by the authors in order to reduce the excessive fragmentation of the base. Secondly, following the information the author included in the field, the institutions were assigned to the macro-institution to they belonged (e.g. School of Dentistry, University of Granada (UGR) and Department of Optics, UGR, are both included within the macro-institution UGR). Similarly, a multi-count approach was followed in the case of collaboration documents between macro-institutions (e.g. a document with authors of UGR and King´s College London was assigned to both institutions). As a general rule, we followed the criteria of the FECYT (http://www.fecyt.es/).

For this study, we used a series of bibliometric indicators. In relation to production, we recorded the total number of documents (NDoc), authors (NAut), institutions, etc., its proportion to the rest of the base, and other involving the relations between the different variables (e.g. articles per journal or articles per institution). Also, we used impact/visibility indicators, including: Impact Factor (IF); the Average IF (AIF of a sample of document is calculated by dividing the sum of the IF of the journal of each document by the total number of documents of the sample); Quartiles (these give the relative position of a journal in each JCR category: Q1, Q2, Q3, Q4); the CAVG or citation average (calculated as the quotient between the number of citation [Ncit] and the number of citable documents, and gives the average of citations reached by the documents, authors or institutions) and the h-index (the number h = number of documents published by the author/institution with at least an h number of citations; e.g. h=30 means the author has 30 articles with 30 or more citations).

## Results

**Figure 1:**
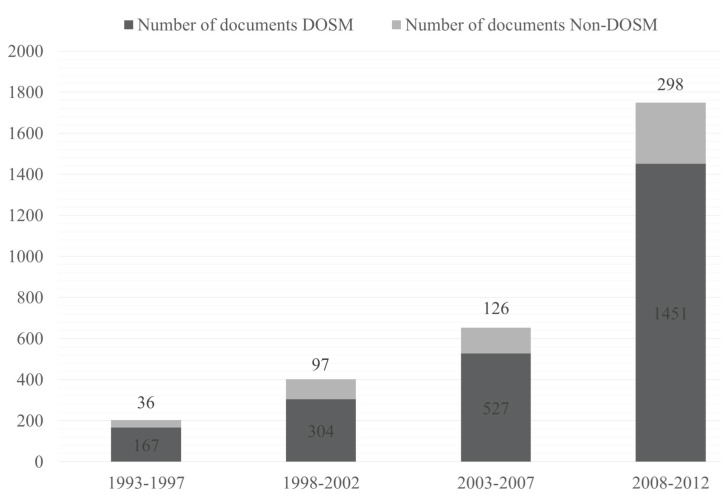
Time course in the number DOSM (Dentistry, Oral Surgery and Medicine) and non-DOSM documents, in absolute terms, during the study period.

The search strategy described enabled us to recover a total number of 3405 documents. Of which we analysed only citable documents, i.e. articles [2718], reviews [209], letters [61], and notes [18], totalling 3006 documents (the 88.3% of total). Of these, 2449 documents (81.5%), were published in journals of DOSM category, while 557 works (18.5%) were in other categories of JCR. (Fig. 1) plots the number of DOSM and non-DOSM documents in the four quinquenniums studied. Related to the impact of this investigation, ( Table 1) shows, in absolute and relative terms, the distribution of the DOSM and non-DOSM production per quartiles in each five-year period studied. Likewise, ( Table 2) shows the 10 most productive non-DOSM categories and also their time course.

**Table 1:**
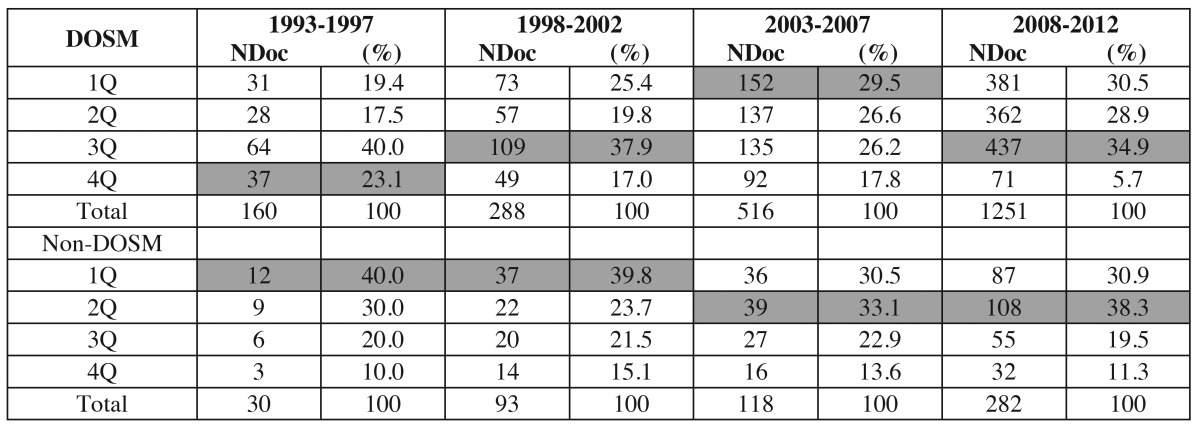
Time course by quinquenniums of quartile distribution in DOSM (Dentistry, Oral Surgery and Medicine) and non-DOSM in absolute (NDoc: Number of Documents) and relative terms (%).

**Table 2:**
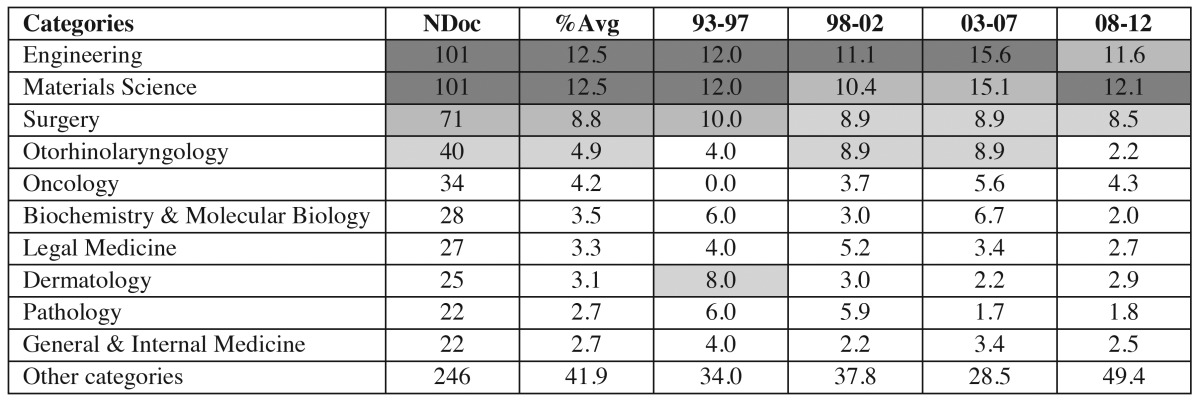
The 10 non-DOSM (Dentistry, Oral Surgery and Medicine) categories of JCR (Journal Citation Reports) with the highest production in the period 1993-2012, and their average proportion (Avg) for the 20 years and for each quinquennium (%). NDoc: Number of Documents.

In relation to the journals, the 3006 documents were published in a total of 380 scientific journals from which 85 belong to DOSM and 395 to the rest of categories. Outstanding in the first group were Medicina Oral Patología Oral y Cirugía Bucal (MOPOCB) with 456 documents, comprising 18.6% of the publications within DOSM and double the second one in rank, Journal of Oral and Maxillofacial Surgery, with 223 (9.1 %). Also with more than 100 documents is the Journal of Clinical Periodontology (106, 4.3%). On the other hand, the non-DOSM production was much more scattered, no journal surpassing 3%. In this group, Journal of Biomedical Materials Research Part A and Part B (Applied Biomaterials) was notable with 14 and 15 documents, respectively.

The quinquennial AIF for the DOSM group were 0.9 in the first one [93-97] and 1.1, 1.6 and 1.8 in the following periods. Therefore we noted a continuous increase in the visibility of the Spanish production, with a surge between the first and the last quinquennium of more than 100%. In the non-DOSM case, the figures for each period were: 1.8, 1.9, 1.9, and 2.6, respectively.

To carry out the thematic analysis of the output, we allocated the documents to specific dental subjects following the process described above.  Table 3 shows the number of documents linked to each specialty and the production percentage of each per five-year periods. It is important to mention that multiple assignations were possible so that the average number of specialties for the periods were: 2.5 in the first one, 3 in the second, 3.5 in the third and 3.8 in the last one.

**Table 3:**
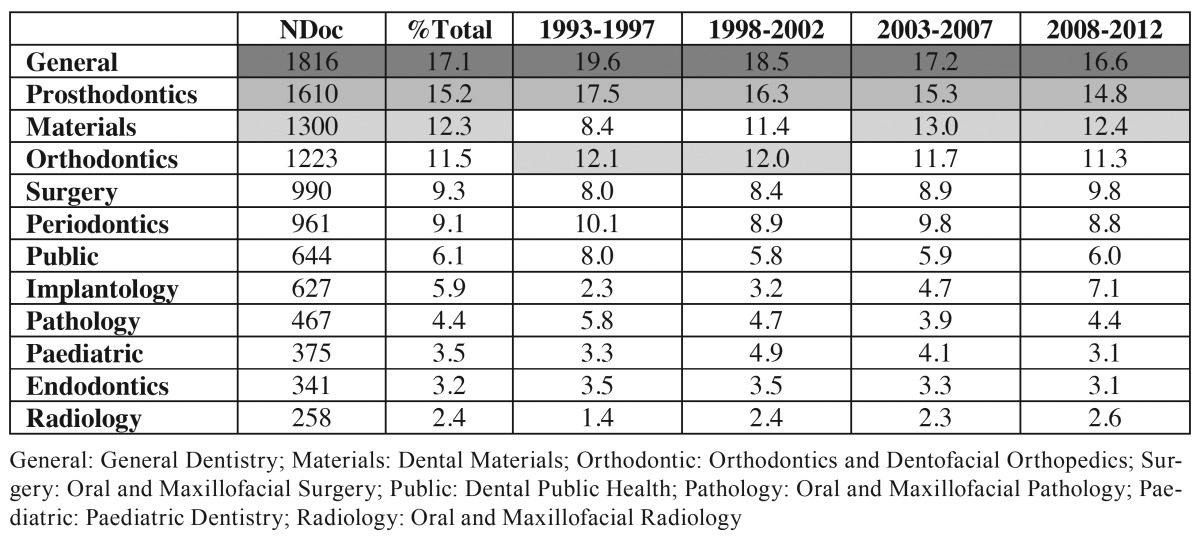
Number of documents (NDoc) per specialty and their proportion of the total, for the whole period and for each quinquennium.

**Figure 2:**
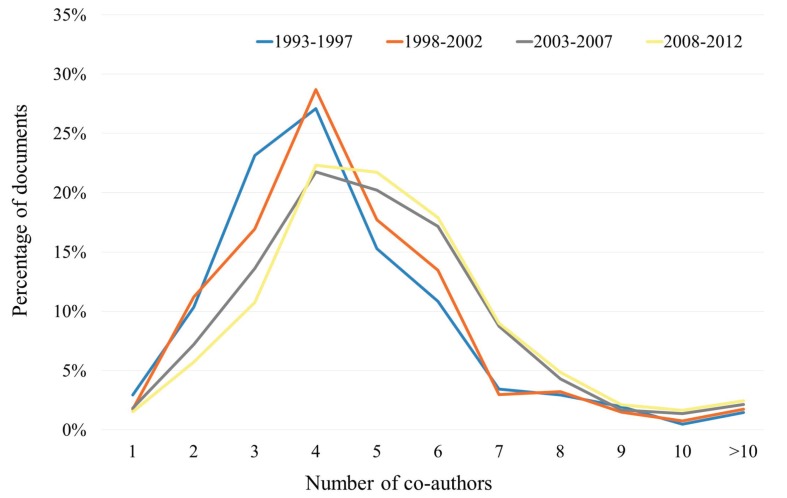
Time course by quinquenniums of the percentage of documents according to the number of co-authors of the overall sample.

Regarding authorship, the most common number of co-authors in the total sample was four (23.4%). Figure 2 shows the time course by quinquenniums of the percentage of documents according to the number of co-authors. The number of authors per document gradually increased, going from 4.7 in the period 1993-97 to 5.3 in 2008-12 period. DOSM and non-DOSM also differed, the average number of authors in the first group being 4.8, and 6.9 in the second. In relation to the most productive authors,  table 4 shows those with 30 or more published documents and the time course for the period. The list includes four authors who did not belong to any Spanish institution and therefore their production is incomplete, reflecting only the documents written in collaboration with Spanish researchers. Also we determined the AIF, the CAVG received for their works, the percentage of documents within first Quartile journals (%Q1), and the h-index (in the foreign cases mentioned above, the h-index is related only to their production in collaboration with Spanish authors).

**Table 4:**
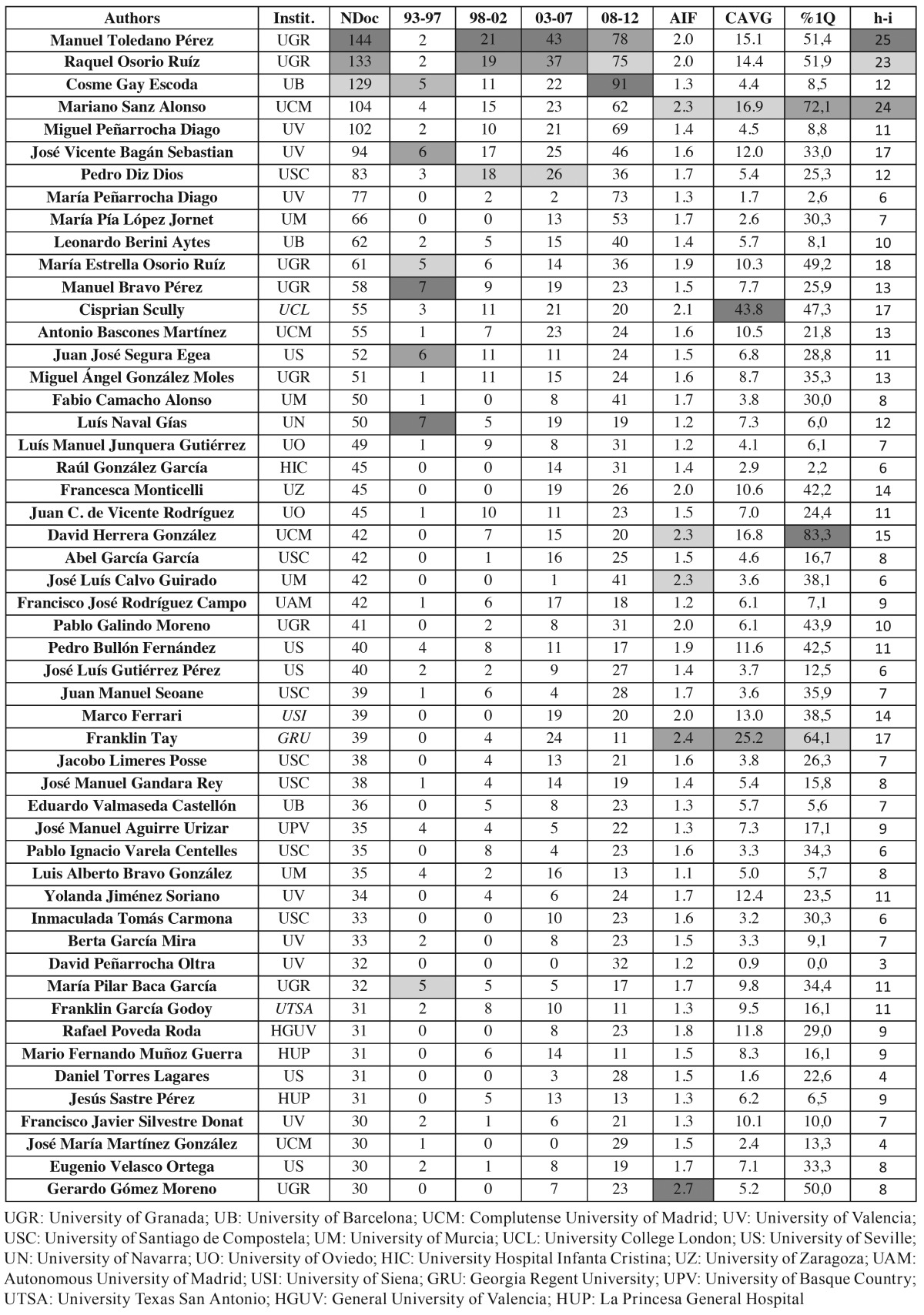
List of authors with 30 or more published documents (NDoc: Number of Documents) in the period 1993-2012 [Total and quinquennium production, Instit. (Institution), AIF (Average Impact Factor), CAVG (Citation Average), %1Q (Proportion of documents in First Quartile), and h-i (h-index)].

Related to institutions, 37 entries in our database contained non-rectifiable mistakes in the field affiliation, so that the analysis covered a total of 2969 documents. We found 1097 producer institutions, of which 311 were Spanish and 786 from the rest of the world. While in the first quinquennium, 155 published some article, while in the last the number reached 827. The proportion of foreign/spanish institutions went from 1.2 in the first period, to 2.3 in the last one.  Table 5 shows the Spanish institutions having the highest production, their time course, the AIF, CAVG received, the percentage of articles in first Quartile (%Q1), and the h-index. The four in the top represent more than half of the production [1592].

**Table 5:**
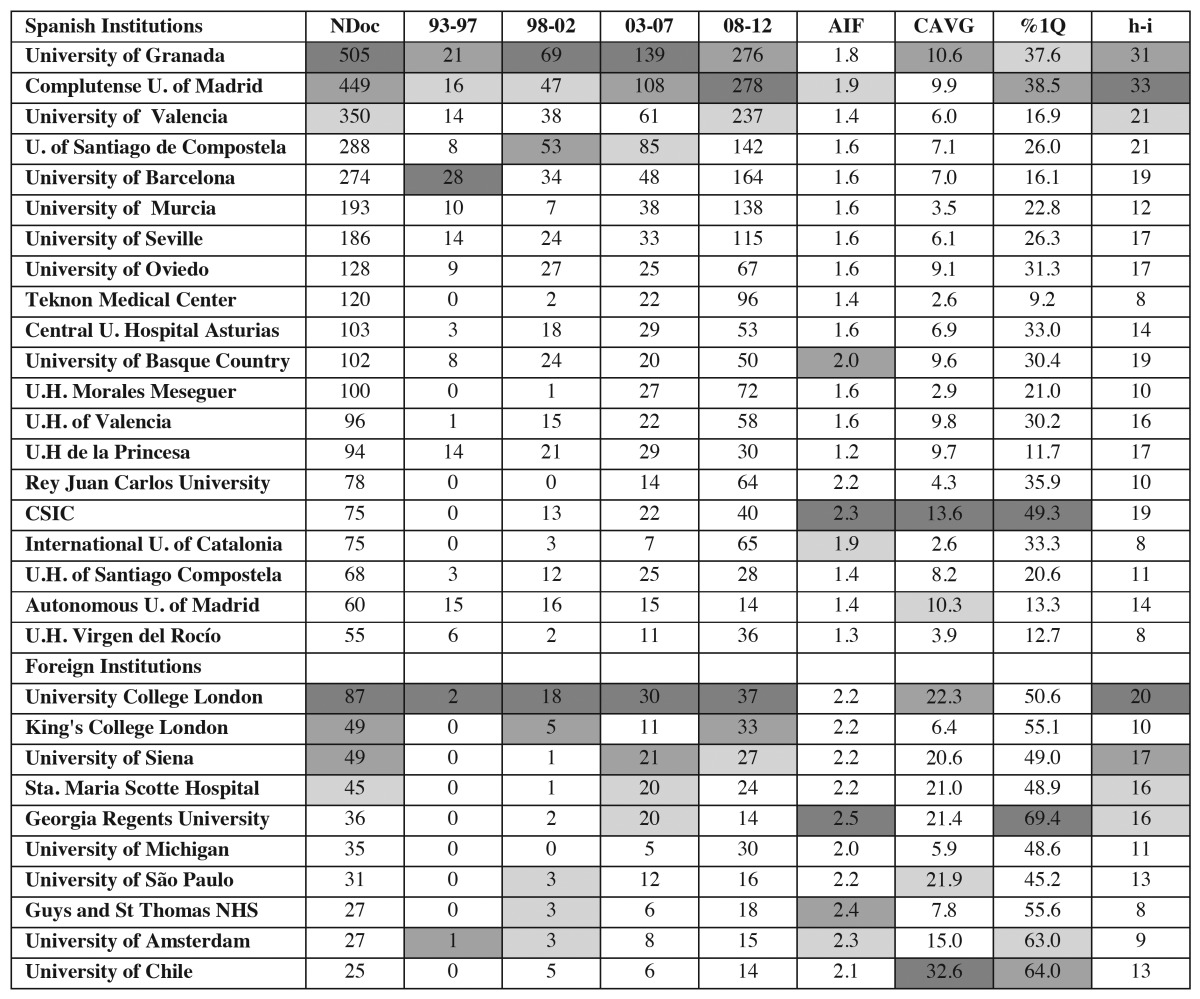
List of the 20 Spanish institutions and the 10 international ones with the highest production during the period 1993-2012 [Total (NDoc: Number of Documents) and quinquennium production, AIF (Average Impact Factor), CAVG (Citation Average), %1Q (Proportion of documents in First Quartile), and h-i (h-index)].

## Discussion

The purpose of the present study is to offer a complete view of the Spanish dental scientific production using the database Thomson Reuters WoS. It is important to remark that we have chosen WoS because it has a big broad coverage of the best journals in the Health Science’s area and also includes information about the quality of the documents such as the impact factor and number of citations.

For a dynamic view, we considered 20 consecutive years and, to compile the most exhaustive possible database, we recovered the documents published not only in the DOSM category but also in the rest of JCR categories. Science today is becoming steadily more multidisciplinary, so that a global analysis of scientific output in Dentistry is necessary (7). These publications in non-dental journals make up 18.5% of the total Spanish production in the period, slightly over the world average, 15% in the 1990s and 17% in the 2000s. It bears mentioning that Spanish authors showed an early interest in publishing outside the DOSM category.

The first point to note in relation to the results is the sharp increase in the number of documents per quinquennium published in Spain in the last 20 years (8.6-fold; Fig. 1). This increase is similar in DOSM and non-DOSM groups (8.7 and 8.3-fold, respectively). The Spanish situation is the opposite of the global one, where the output increased more in non-DOSM (1.5-fold) than in DOSM (1.3-fold) in the last two decades (7). The rise in the number of published documents could be ascribed to the growing number of journals in the base, but under this assumption the increase in the Spanish production would be equivalent to the one undergone by Dentistry in the rest of the world. Pulgar *et al*. (7) established that the absolute growth in the number of documents was caused by the increase in the number of journals in the category, so that the weight of the area in the database did not change. If Spanish production would have followed the same trend, the increase would have been 1.3-fold, whereas the figure for Spain is 8.6, demonstrating a real increase in the Spanish production weight in international dental research.

Although the Spanish production improved quantitatively, from a qualitative standpoint, the tendency is not so clear.  Table 1, indicates that the number of documents published in Q1 and Q2 DOSM journals grew between the first and third quinquennium, going from 36.9% to 56%; however, in the last period, although the sum of Q1 and Q2 was 59.5%, the journals in Q3 received a higher number of articles due to the inclusion, as we will analyse below, of the journal Medicina Oral Patología Oral y Cirugía Bucal (MOPOCB). In the case of non-DOSM publications, the situation changed from publishing mostly in Q1, in the first and second quinquennium, to publishing in Q2 in the following periods. Consequently, our results imply that the impact of the dental Spanish publications has stabilized in the last quinquennium and is strongly affected by the appearance of MOPOCB in DOSM. Finally, there was the progressive increase in AIF, 100% in DOSM, and 44% in non-DOSM.

Outside DOSM, the categories Materials Science and Engineering are ranked in first position with 12.5% of the documents. They are followed by Surgery with 8.8%, and the three keep their position throughout the period. The rest of categories that appear in  Table 2, though they maintain their presence over 2%, more or less markedly decline during the time period, signifying an increase in “other categories” from 34 to 49.4. This could be interpreted as a higher diversification in the Spanish dental science in JCR categories.

In relation to publications, in DOSM, the journal MOPOCB tops the list with 456 documents. This journal was indexed for the first time on 2008 and despite being on our list for only five years, it reached the highest position in the ranking of most productive journals. This was because the journal is edited in Spain and is the only Spanish publication about dentistry that appears on the JCR, a situation that has helped to improve the visibility of Spanish dentistry in WoS databases. There is no doubt about this, given that, according to our data the documents published in MOPOCB comprised 31.4% of all publications in the entire five-year period in DOSM and the 26.1% of total. This confirms that the geographical location of the publications influences the choice of the journal by the authors in order to publish. Camí *et al*. (8) described a similar situation, also in Spain, when the journal Medicina Clínica was first indexed on Science Citation Index. MOPOCB is followed by Journal of Oral and Maxillofacial Surgery and Journal of Clinical Periodontology, with 223 and 106 documents, respectively; these three publications represent 26.1% of Spanish production.

The specialty with the highest presence is General Dentistry with 17.1% of the documents ( Table 3). It is followed very closely by Prosthodontics with 15.2% and, in the third place, with almost the same percentage, Dental materials and Orthodontics and Dentofacial Orthopaedics, at around 12%. These four categories represent 56.1% of total. If we compare the first and last quinquennium the most remarkable trend is the rise of Implantology and Dental Materials at the expense of the two in the top for the whole period and, although they keep their position, the margin is smaller. The previous work by Pulgar *et al*. (7) demonstrated by means of analysing social networks, the growing interconnection between the dental specialties. Our data suggest a similar phenomenon, since the average of specialties to which the documents belong increased from 2.5 in the first period to 3.8 in the last, perhaps because of the more interdisciplinary nature of the works.

Regarding to authorship, there is a continuous increase in the average number of co-authors per document over these 20 years, as reflected in figure 2. This trend matches the one that world Dentistry has in DOSM, but not in non-DOSM (7), since the average number of collaborators is higher in the first period than in the rest. It has been speculated that a higher number of co-authors could be related to the necessity of including more researchers in teams when the objective is to publish outside DOSM, i.e. multidisciplinary teams where dentists and specialists in other areas work together (7).

The two top producers in our list belong to the University of Granada. They are Toledano M. and Osorio R. with 144 and 133 documents, respectively. They are followed by Gay C. with 128 from the University of Barcelona. Only these three authors plus Sanz M. from the Complutense University of Madrid [104] and Peñarrocha M. from the University of Valencia [102], reached 100 documents during the study period. In general, the most remarkable trend was the sharp increase in productivity in all the authors over the 20 years. On the other hand, the universities of Granada and Valencia were the institutions with a largest presence among the most productive Spanish authors (7 each one). They were followed by the universities of Santiago and Seville, with 6 and 5, respectively. Only three institutions were hospitals instead of universities: General University in Valencia, De la Princesa in Madrid, and Infanta Cristina in Badajoz.

The highest-quality indicators are irregularly distributed and it is possible to find authors with high production associated with good AIF (Toledano M., Osorio R.and Sanz, M.) while others are quite under average. It is difficult to contextualize the AIF, since it is considered the global production of the author in both DOSM and non-DOSM. However, the %Q1 offers a better valuation of the expected author impact, and the great variability of this parameter in our series. Otherwise, regarding CAVG, the article by Pulgar *et al*. (7) provides data to compare the value that this indicator reaches in our work. In the three trienniums analysed, these authors calculated a CAVG of 8.93 in DOSM and 11.15 for non-DOSM; in our case, working out the average for both groups for the most productive authors gave a similar value although slightly lower (8.1). Nevertheless, while the AIF depends on the journal in which the authors have published, the CAVG is an objective datum on the visibility and impact of an author’s production in the international context.

One of the most striking facts of our study in the great increase in Spanish production. This could be explained by the growing number of actors involved. There has been a substantial increase in institutions, from 155 during the 1993-97 period to 827 in 2008-12. There is also a jump in the number of authors, both in absolute numbers as well as in their individual productivity: in the first period, 31 authors published 90 documents while in the last, 50 worked on 1611 documents. Therefore, the rise in Spanish scientific output was favoured by greater collaboration, both from authors as well institutions. This aspect of scientific activity will be the object of a next work.

The 20 Spanish institutions with the highest production were identified together with the 10 main foreign institutions with which they collaborate. Most striking are the universities of Granada and Complutense of Madrid, both with a similar trend over the entire period ( Table 5), and the University of Valencia, which increased their production fourfold from the third to fourth quinquennium. Remarkably, the first eight institutions are public universities, and the ninth is the first hospital, Teknon Medical Center, which has private funding. In relation to international collaboration, the countries most connected to Spanish institutions are USA, Italy, UK, and Germany, and the most productive institution linked to Spain is University College London, also situated between the 20 most productive with 87 documents. The best visibility marks, although not being in the first positions in absolute production, belong to CSIC, which ranks substantially ahead of the rest and followed by the two biggest producers, the universities of Granada and Complutense of Madrid. Nevertheless, perhaps the most relevant fact is that foreign institutions have better values in these indicators, implying that Spanish institutions and their authors look for international collaboration to improve the quality of their output.

To summarize the results, Spanish dental research is no longer occasional, with only a few authors involved and low international impact. Now the situation is similar to the rest of medical specialties in Spain and, because the amount of production and its impact, it shows the degree of maturity reached in the last 20 years, although certain peculiarities remain, such as the university being the most productive institution.
